# Using Aerial and Vehicular NFV Infrastructures to Agilely Create Vertical Services

**DOI:** 10.3390/s21041342

**Published:** 2021-02-13

**Authors:** Borja Nogales, Miguel Silva, Ivan Vidal, Miguel Luís, Francisco Valera, Susana Sargento, Arturo Azcorra

**Affiliations:** 1 Telematic Engineering Department, Universidad Carlos III de Madrid, Avda. Universidad, 30, 28911 Madrid, Spain; bdorado@pa.uc3m.es (B.N.); fvalera@it.uc3m.es (F.V.); azcorra@it.uc3m.es (A.A.); 2 Instituto de Telecomunicações, 3810-193 Aveiro, Portugal; maps@ua.pt (M.S.); nmal@av.it.pt (M.L.); susana@ua.pt (S.S.); 3 ISEL-Instituto Superior de Engenharia de Lisboa, Instituto Politécnico de Lisboa, 1959-007 Lisboa, Portugal; 4 Department of Electronics, Telecommunications and Informatics (DETI), University of Aveiro, 3810-193 Aveiro, Portugal; 5 IMDEA Networks Institute, Avda. del Mar Mediterráneo 22, 28918 Madrid, Spain

**Keywords:** small unmanned aerial vehicles (SUAVs), flying ad hoc networks (FANET), vehicular ad hoc networks (VANET), network functions virtualization (NFV), orchestration, vertical services, network slices

## Abstract

5G communications have become an enabler for the creation of new and more complex networking scenarios, bringing together different vertical ecosystems. Such behavior has been fostered by the network function virtualization (NFV) concept, where the orchestration and virtualization capabilities allow the possibility of dynamically supplying network resources according to its needs. Nevertheless, the integration and performance of heterogeneous network environments, each one supported by a different provider, and with specific characteristics and requirements, in a single NFV framework is not straightforward. In this work we propose an NFV-based framework capable of supporting the flexible, cost-effective deployment of vertical services, through the integration of two distinguished mobile environments and their networks: small sized unmanned aerial vehicles (SUAVs), supporting a flying ad hoc network (FANET) and vehicles, promoting a vehicular ad hoc network (VANET). In this context, a use case involving the public safety vertical will be used as an illustrative example to showcase the potential of this framework. This work also includes the technical implementation details of the framework proposed, allowing to analyse and discuss the delays on the network services deployment process. The results show that the deployment times can be significantly reduced through a distributed VNF configuration function based on the publish–subscribe model.

## 1. Introduction

Over the last few years, the transition to the new generation of mobile communications (i.e., the fifth generation, or 5G) has been gradually taking pace. To reach this current stage, the research community has carried out an arduous exercise to first define the requirements to be fulfilled by this new generation in order to address the direction taken by society’s use of information technologies, and then, to establish the basis in the form of standards to be followed when developing the new generation technologies. In both cases, the research activity developed under the scope of projects funded by the European Union (EU), such as those corresponding to the different phases of the 5G infrastructure public private partnership (5G PPP) initiative, or programmes such as the EU programme horizon 2020, have played a very significant role [1]. This is due to the fact that during the course of these projects, numerous synergies arise between different research entities that allow both the achievement of the requirements defined for this new era of communications, and the identification of the forthcoming aspects to be addressed.

One of the most relevant challenges studied by these research activities is focused on realizing a paradigm shift concerning the model followed by the previous generations of mobile networks for the provision of communication services. This paradigm shift considered the 5G aims to enable the transition from a model in which the services were oriented to satisfy the human communications (referring to human communications in terms of voice/video calls, data transmission, and Internet access) regardless of the necessities of the end users, towards a model in which the communication services provision entails the adoption of the particular requirements exhibited by different industry sectors or verticals [2,3]. Thus, 5G will contribute to a global digital transformation, involving diverse vertical sectors such as automotive, smart cities, healthcare or public safety among others, to support the creation of an innovative ecosystem capable of accommodating advanced and modern developments in both technical and business domains. This novel ecosystem will bring a more comprehensive portfolio of services and applications with a resulting multiplicity of requirements beyond the current voice and mobile broadband, encompassing the massive connection of machine-type devices, high reliability, ultra-low latency and an enhanced mobile broadband with higher bandwidth [4,5,6]. Nonetheless, the full realization of this vision within the next generation of mobile network is challenging due to, among other reasons, the lack of flexibility to integrate heterogeneous network infrastructures capable of supporting the cost-effective deployment of vertical services tailored to a particular set of requirements.

From this perspective, the intersection of two research lines has led to the work covered here. On the one hand, headed by the Universidad Carlos III de Madrid, one of these lines tackles the study of the feasibility to provide communications services using Small sized Unmanned Aerial Vehicles (SUAVs) in areas where no telecommunications infrastructure is present, or it is unstable (e.g., in rural areas or in areas devastated by emergency incidents). This is possible because of the intrinsic ability of these vehicles to be positioned in almost any location, even in those that are hard-to-reach. Notwithstanding the high expectations placed on the SUAVs for their participation in the near future networks, one of the most notable weaknesses of these devices is the lack of flexibility in terms of serving a wide range of functionalities and their adaptation to the requirements imposed by particular interests of each occasion. This is because the design and implementation of the SUAVs are usually aimed at addressing a unique, specific-mission task (e.g., collect thermal images, provide a communications relay, or surveillance). For this reason, and with the aim of catering this gap in the SUAV’s development, the core of this research has been focused into endowing SUAVs with the ability of adopting the virtualization paradigm introduced by the network function virtualization (NFV) technology [7,8,9,10].

On the other hand, the University of Aveiro along with the Instituto de Telecomunicações have aimed one of their research interests to the vehicular networks, and how offering different applications and services to be used by the vehicles and/or their occupants within the scope of the vehicular network. The main objective of this research is to deploy different functionalities inside the vehicles themselves, as close as possible to the end users, through the utilization of the NFV technology. With this, it is possible to allow end users to access specific services, even when vehicles are in situations of intermittent connectivity with the vehicular infrastructure, as well as to allow less communication delays for critical services, such as road safety services, by bringing these functions closer to end users [11,12,13].

Taking into account the above considerations, in this paper we present the definition and implementation of a comprehensive framework capable of integrating dynamically heterogeneous NFV infrastructures distributed across different geographical locations in order to support the deployment of elaborated vertical services. Specifically, this paper analyses how to carry out the integration between three NFV infrastructures with clearly differentiated capacities. In the first place, the framework envisages the integration of an infrastructure with high computing resources that allows the development of virtualized network functions (VNFs) that can be spanned within a core domain of a 5G network. With this approach, the 5G Telefonica Open Network Innovation Centre (5TONIC) laboratory [14], located in Madrid (Spain), is extended and used as the core infrastructure. This laboratory also includes a set of SUAV devices to enable the development of the research line mentioned above, which aims to provide these mobile devices with the ability to support the NFV technology, and thus dynamically assist in the deployment of new generation networks, without incurring the limitations usually entailed by these devices to offer different functionalities. This leads us to the second infrastructure contemplated in this work and that will incorporate into the framework the ability of destining on-demand the deployment of services in a flexible and automated manner wherever it is required, leveraging for this the inherent mobility capacity of this type of devices. In addition, the final infrastructure covered by the framework is an automotive environment capable of deploying opportunistically functionalities on real vehicles connected both with vehicle-to-vehicle (V2V) and vehicle-to-infrastructure (V2I) communications, so that end users in those vehicles may have enhanced access to the possible services to be deployed. In this case, the Instituto de Telecomunicações [15] located at Aveiro (Portugal), makes available the necessary resources that have been used to performed the implementation of this part of the framework. Last but not least, it is worth mentioning that this work contemplates the integration of each and every one of the mentioned infrastructures under the same MANO NFV stack, which places the different components that make the stack up in a distributed way, alongside each infrastructure.

By means of this innovative joint integration, services and applications for specific verticals can be supplied. In this sense, and with the objective of corroborating the practicality of the proposed framework, the work includes the definition of a scenario pertaining to the public safety vertical that aims to monitor the state of a road given that possible adverse situations are foreseen. Namely, due to an expected increase in the number of vehicles crossing a road due to an exit operation caused by a holiday period, or to monitor the so-called black spots (road sections where the number of accidents is accentuated) during the peak hours of the day. In addition, this scenario also considers the occurrence of an event originating an emergency situation (e.g., a collision between vehicles), to show the ability of the framework to flexibly adapt the deployment to handle this unexpected delicate situation. From this perspective, the work also includes the development of a novel solution of a configuration function based on the publish–subscribe model that can be incorporated into the MANO stack to address the agile configuration of the functionalities to be deployed in this type of emergency situation, since through the experimentation results it has been detected, as a missing aspect, that the default implementation did not properly manage this configuration task. The configuration function approach is then able to significantly reduce the deployment times to the ones feasible in an emergency scenario. Moreover, the services being provided through the different infrastructures can be of high-bandwidth and quality, with different video streams transmitted simultaneously.

This article is the result of a collaborative work that combines two different research lines. In summary, this work includes the following main contributions:

- It presents the definition of an NFV framework capable of integrating aerial and vehicular NFV infrastructures, to enable the cost-effective and flexible deployment of vertical services.
- It includes the detailed description of the realization of a public safety vertical use case, to emphasize the practicality and potential benefits of the proposed framework.
- It presents the integration of two remote NFV infrastructures: an infrastructure of SUAVs, which can be deployed on demand, and an automotive infrastructure, supporting the opportunistic provision of services. Both infrastructures are provided by research groups of different countries (Spain and Portugal).
- It considers the network slicing model as a design key, to exploit the ability of modifying the services in real time, in the most agile and efficient possible manner.
- It provides the implementation details of both the framework and the network services, through the use of open-source technologies.
- Finally, it includes a novel solution based on the publish–subscribe model to agilely carry out the configuration of VNFs, significantly reducing the deployment times in an emergency scenario.

The rest of this article is structured as follows. Section 2 studies the current state of the art in this research field. The main conceptual aspects to be considered throughout the definition of the framework to support the flexible deployment of vertical services, encompassing distinct NFV infrastructures, in which the computational resources are provided by mobile devices, are addressed in Section 3. Section 4 delves into the applicability of the framework proposed by means of defining a significantly complex scenario, with its main architectural blocks detailed, emphasizing the practicality and potential benefits of the proposed framework, while Section 5 depicts the evaluation results. Finally, Section 6 presents the conclusions of the work performed and includes the directions for future work.

## 2. Background and Motivation

The advances on the new generation of mobile networks, the fifth generation (5G), have motivated new paradigms and technologies for the vehicular communications to emerge, such as the case of the Cellular Vehicular to Everything (C-V2X), allowing faster communication with a greater variety of entities than it was possible before. In this new generation of mobile networks, VANETs are seen as one of the central blocks that make possible the idea of always being connected and able to communicate, given that they can be seen as a connectivity on the move. Considering these possibilities in terms of communication, VANETs allow a wide range of applications and uses within 5G networks, due to the simple and easy ability to share information between the vehicles themselves and the infrastructure [16].

Another aspect that is becoming more and more popular is the shift to a greater use of software-defined networks [17,18]. One of the most important concepts in this area is the concept of using virtualization technology to help decouple software from hardware, by virtualizing entire network functions, previously carried out in specific hardware, into software blocks which can later be deployed throughout the network to create complex services. This is the main concept of one of the new core technologies of 5G, called network function virtualization [19], which provides many benefits, such as greater flexibility and scalability [20,21].

One of the latest research topics of interest to many researchers is how to bring the new concepts of network virtualization and cloud technologies into a more dynamic environment such as vehicular networks. Hussain et al. [22] proposed potential architectural frameworks for different types of cloud scenarios in VANETs. The authors present a division into three categories: vehicular cloud (VC), vehicles using clouds (VuC), and hybrid vehicular clouds (HVC). VCs can be seen as groups of vehicles providing their resources in order to enable cloud services (e.g., cars parked in parking lots). As far as the VUC is concerned, these operate on the basis of interconnecting traditional clouds with VANETs, allowing the users of the VANETs to use the services provided by the cloud. Finally, we have HVC which is a combination of the two previous frameworks, VC and VUC, where the vehicles provide their resources to the cloud while at the same time being able to use its services.

A solution that falls within the scope of VCs and that demonstrates the use of vehicle resources to support cloud services is the solution presented by Zingirianet et al. [23], where the authors developed a cloud composed only of vehicles. The cloud works under a new experimental service modality for vehicle platforms, called Sensor-as-a-Service (SenaaS), which gives third parties access to the devices and sensors present on the vehicles. This means that third parties can then take advantage of these resources to create various types of applications and services, such as vehicle monitoring applications.

One work that bases itself on the concepts presented so far, is the solution proposed by Zhu et al. [24] where the authors have brought together the concepts of cloud and NFV into a single framework. Nowadays, most of the vehicles launched by automotive companies are accompanied by complex systems, called intelligent onboard system (IOS). This type of systems is interesting because they allow vehicles and their occupants to benefit from certain types of services (e.g., location-based services) in a simple manner. The main problem with this type of systems is that they have a closed architecture, which means that, updating or even supporting new types of services becomes more difficult. In this work, the authors focused around creating a framework for NFV-based VANETs that aims to make these IOS systems a more open platform, and thus allowing them to host several different types of services in a simpler and more dynamic way. The work presented in [11] focuses on the concept of extending the cloud to the edge of a VANET. This work develops a solution that makes possible to easily deploy flexibly network services in a vehicular network, focusing on using the hardware already present on the vehicles, developing a more open and flexible solution which is capable of using various types of hardware platforms.

On the other hand, the key role reserved for the UAVs within the deployment of the new generation networks is no longer a mere proposition from the academic community, but one of the undeniable factors in this new era of communications. Indeed, much work is being done to define which benefits can be brought from these particular devices to the current 5G communications systems for provisioning different services or applications. In this context, authors in [25] present the wide range of services and applications that can be assisted by this sort of devices, in addition to analysing what are the main challenges that need to be addressed in order to support the integration of the aerial vehicles into 5G systems and beyond. Following this theme, the authors in [26] present an architecture with the aim of satisfying the requirements of the fifth generation of mobile networks applications through the use of aerial nodes that offer an aerial access network where numerous users and devices compete for connectivity and data rates. In [27], the authors study the use of UAVs to extend the 5G networks and thus, ensuring ultra-low latency in processing data for delay-constrained applications.

In any case, the trend noted in the literature shows that the research community has a strong focus on targeting mobile ad hoc networks (both VANETs and FANETS) to address the major challenges imposed by 5G networks.

## 3. Description of the Aerial and Vehicular NFV Framework

**Figure 1:**
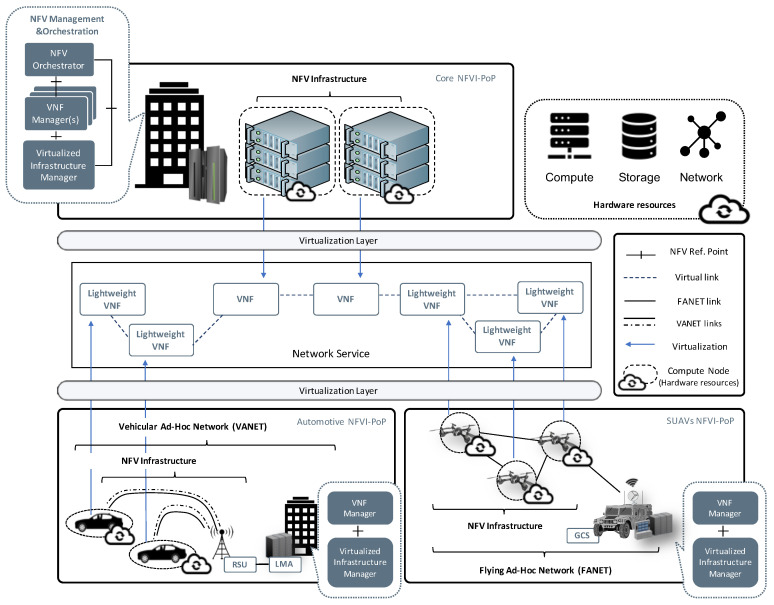
Overall framework architecture.

Inspired by the prior work independently carried out in both the SUAV and automotive environments in order to support the flexible and automated deployment of network services through the use of the NFV technology, this work aims to define an overall framework, aligned with the design principles of the NFV architectural framework published by ETSI [28], to enable the creation of a more complete and distributed NFV ecosystem, supporting the flexible incorporation of diverse NFV infrastructures from distinct service providers, distributed across different geographical locations. Thus, being able to host complex communication services and applications. In this context, Figure 1 graphically shows the design bases of the entire ecosystem.

The ecosystem consists of three network function virtualization infrastructure points of presence (NFVI-PoPs), where the needed resources are available to support the deployment of virtualized network functions (VNFs) that, through their interactivity, will result in the provisioning of different network services. First, the upper section of Figure 1 represents the so-called Core NFVI-PoP with an NFV infrastructure (NFVI) with high availability of resources in terms of computing, network and storage to accommodate services corresponding to the core network domain. Alongside with this NFVI, the framework places part of the management and orchestration (MANO) stack in charge of orchestrating and managing the deployment of the services that will be hosted within the whole ecosystem. On this basis, the Virtual Infrastructure Manager (VIM) block within the MANO stack addresses the management and coordination of both hardware and virtual resources of this first NFVI-PoP. In conjunction with VIM, the NFV orchestrator (NFVO) and the VNF manager (VNFM) are also located in the mentioned MANO stack. Such VNFM is in charge of supporting both the configuration of the VNFs hosted by the Core NFVI-PoP, and their lifecycle management, to provide to each virtualization unit with the expected functionality within a network service. For its part, the NFVO encompasses the lifecycle management of the network services, specifying how VNFs are connected to one another to form a network service, and triggering their deployment or depletion when it is required.

Up to this point, it has been simply described the part of the framework corresponding to a regular NFV system. Below, and in more detail, it is described how such system can be extended with two additional NFV infrastructures in which computation capabilities are provided by mobile devices, in particular, using SUAVs and vehicles.

### 3.1. The SUAV NFV Infrastructure

The lower right corner of Figure 1 reflects the component of the framework referred to as SUAVs NFVI-PoP, following a conceptual overview in which every SUAV device comprises a computational unit that offers its hardware resources in terms of computation, storage, and networking, with the aim of enabling the execution of VNFs. It is worth noting that these resources are mainly limited due to the compact size of the SUAVs, which also implies that they cannot carry any complementary hardware platform to greatly increase their computational capabilities to not compromise the flight operations. Due to this limitation on the available resources, the softwarization units (referred to as lightweight VNFs in the figure) have to implement their functionality in such a way that their execution does not involve a significant cost in terms of computing. Thus, endowing SUAVs with the ability of adopting the virtualization paradigm introduced by the NFV technology, allows to provide an alternative, on-demand communication infrastructure wherever it is needed, either in areas where telecommunication infrastructure is not provided, or insufficient (e.g., rural areas or areas severely damaged because of an emergency). This alternative also provides a high degree of flexibility when serving a wide range of functionalities, and their adaptation to the requirements imposed by particular interests of each occasion.

In this context, the VIM component, located in the ground control station (GCS), coordinates the integration of different SUAV units within the computing platform with the aim of comprising the SUAVs NFVI-PoP as shown in Figure 1. In addition, this block is in charge of coordinating the available hardware resources and allocating them to the virtual resources that will fulfil the computing, storage and networking requirements of each lightweight VNF deployed into this infrastructure of the framework. The integration of this element with the MANO stack located in the Core NFVI-PoP enables the framework to orchestrate multi-site services in which both NFVIs will accommodate different VNFs or lightweight VNFs capable of interoperating among themselves, with the added advantage that the SUAV-based infrastructure can be positioned wherever required or desired. Furthermore, the framework considers the possibility of including a VNFM element located in the GCS next to the VIM, so that, in case that the interoperation with the MANO stack of the Core NFVI-PoP is interrupted, the lifecycle of VNFs executed by the SUAVs NFVI-PoP can continue to be managed. Finally, the implementation of the flying ad hoc network (FANET) represents a critical element to ensure the proper operation of the SUAVs NFVI-PoP. The purpose of this component is threefold: (1) to enable the communications between the SUAVs and the VIM to allow the latter to coordinate the operations regarding the NFVI (i.e., manage the hardware and virtual resources); (2) to provide the underlying substrate on which the virtual networks will be created by the VIM in order to interconnect the VNFs and thus support the communications that will determine the functionality of the network service; (3) and to allow the automated configuration of the VNFs, supporting the communications from the VNFM with the virtualization units once these have been provisioned by the VIM.

### 3.2. The Automotive NFV Infrastructure

Figure 1 also depicts, in the lower left part of it, the last component of the overall framework, referred to as Automotive NFVI-PoP. In this case, the NFV technology enables the deployment of lightweight virtual network functions within vehicular networks (indeed, inside the vehicles themselves), as close as possible to the end-users. This makes possible end-users to access to particular services, even when vehicles are in situations of intermittent connectivity with the vehicular infrastructure, as well as to allow less communication delays for critical services (e.g., road safety services) by bringing these lightweight virtual functions closer to end-users with the help of general purpose hardware platform deployed on the vehicles.

**Figure 2:**
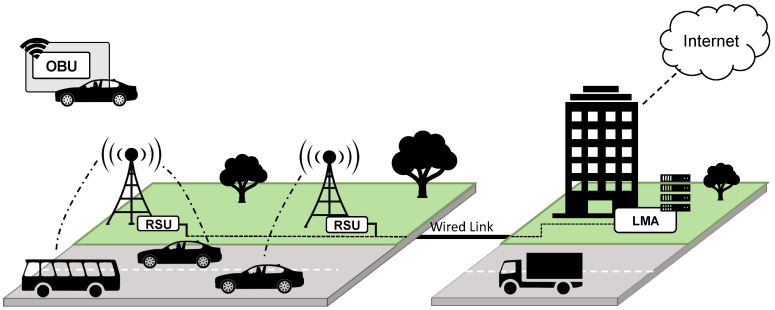
Simple representation of a N-PMIPv6 based VANET, illustrating only its main elements.

In this part of the framework, the vehicular network used is a VANET with mobility and multihoming support, based on the Network-Proxy Mobile IPv6 architecture (N-PMIPv6) with the addition of certain mechanisms that were developed to support transparent handovers and simultaneous multihoming [11]. Figure 2 shows a simple representation of this type of VANET, showcasing its main components and how they are connected. The local mobility anchor (LMA) is the home agent of all the mobile nodes, and it is the responsible for managing the network’s communications; the road side units (RSUs) are the mobile access gateways which are connected to the LMA and provide connection points for on-board units (OBUs); lastly the OBUs play the role of mobile routers, and being placed inside the vehicles, they are in charge of providing connectivity to end-users with the remaining elements included in the vehicular network. The LMA is also the element that connects the vehicular network with the Internet, and consequently, with the NFV orchestrator.

Taking into account the vehicular network used in this work, an extra hardware platform alongside the OBUs is used to host the virtualized functions. This is due to the fact that the computational unit present in the vehicles to enable the VANET related operations (the OBU), was conceived to solely run the VANET software, and it does not have the ability nor the resources to support the virtualization required by the NFV technology. From this perspective, the aim of this infrastructure is to supplement an existing VANET with the capabilities to effectively support the NFV technology, involving as few changes as possible in the nature operation of the VANET, and in its structure. To accomplish that, its corresponding VIM and VNFM blocks are located outside the scope of the VANET, but in a way that they can still communicate with the elements encompassed by the vehicular network. This hardware is connected to the OBUs and comprises the NFVI in the Automotive NFVI-PoP, thus enabling the execution of the lightweight VNFs. In this sense, and in the same way as with the SUAVs-based infrastructure, the integration of the VIM and VNFM elements addressing the resource management (both hardware and software) of the automotive NFVI-PoP, in conjunction with the MANO stack located in core NFVI-PoP, allows the overall framework to orchestrate sophisticated communication services across the three computing infrastructures. In this case, the potential benefit that includes the vehicle infrastructure is to make possible to coordinate the execution of virtualized functions near to the vehicle members in a flexible and automated manner.

## 4. Use Case Description

In the following section, the application of the proposed framework to a particular use case is defined, whose goal is to highlight potential scenarios for its implementation, as well as emphasising the significant benefits of such implementation. We will start with the use case description, and then we will describe the details to be considered for the further implementation of the network service.

### 4.1. Initial Vertical Service Deployment

With the beginning of the new era of mobile networks (or 5G, as it is commonly referred), an immense number of connected devices is envisioned to play an important role in the provision of application services to end-users. In particular, the automotive arena is considered as one of the most clearest examples in which the challenges specified for this new generation of communications (e.g., reduced latency, high bandwidth or reliability, low energy consumption, etc.) need to be accomplished. Accordingly, the industry and the research community have presented how the technology improvements could be used to enable a secure, connected and automated driving [29].

**Figure 3:**
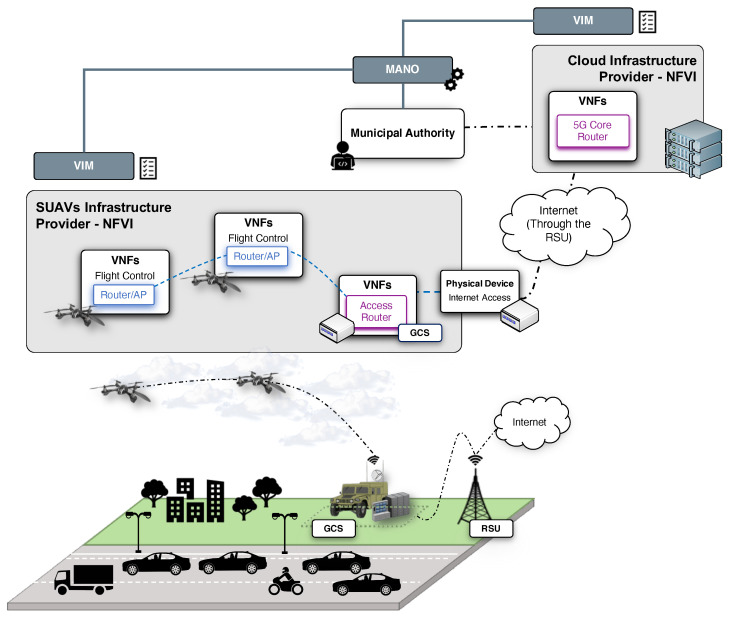
Overview of the initial service provided by the SUAVs deployment.

In this context, the proposed use case considers a common situation where dense road traffic conditions can be expected in advance, e.g., a traffic jam in a major highway at the beginning of a holiday period. In this situation, a flying network of SUAVs (FANET) with NFV capabilities can be deployed by a public safety department of a municipal authority with the aim of improving the situational awareness of the road conditions. As depicted in Figure 3, a SUAVs infrastructure provider supplies to the municipal authority with a set of these SUAVs composing one of the NFVI-PoPs introduced in the previous section (see Section 3.1). These SUAVs build a flying ad hoc network over the motorway infrastructures, so that, through the execution and interoperation of different softwarization units or VNFs, a network service to monitor the situation on the road is enabled. For this purpose, the municipal authority coordinates the deployment of such service by making use of the MANO stack present in the overall platform. Other SUAVs are in charge of collecting relevant information (i.e., video and images). The information produced by the SUAVs and the vehicles is delivered to the public safety department through the mentioned network service, traversing the aerial network comprised by the SUAVs and the terrestrial communications infrastructure provided by the RSUs, facilitating decision-making processes (e.g., the platform could be used to verify that the predictions of traffic flows are fulfilled, and modify the strategies to address the road conditions). In addition, this may complement the resources of cellular access networks serving the users, and thereby preventing a potential stage of congestion caused by dense road traffic situations.

Moreover, the aerial network can be used to support the dissemination of relevant information from the municipal authority to users at cars, making also use of relay VNFs deployed on cars and SUAVs. This will enable new types of applications that take advantage of the availability of traffic and driving information, as suggested in [29].

### 4.2. Creating an Unheralded Vertical Service

**Figure 4:**
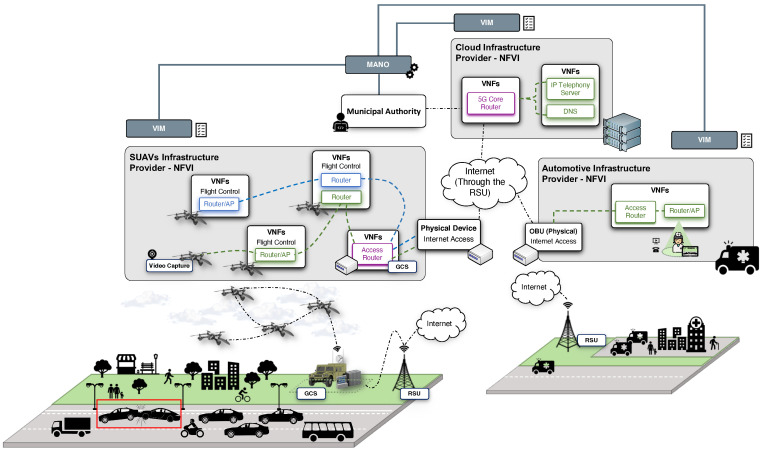
Emergency situation: a complementary network service is deployed to handle the emergency.

One of the main benefits of deployments, such as the one presented above, is the ability to be adapted in a versatile and expeditious manner in order to address the emerging demands imposed by the altered circumstances. For instance, if an emergency occurs, the depicted deployment can integrate additional SUAVs to extend the offered functionality, modify the trajectory of existing ones, execute new functionalities or update the existing ones, and even to release allocated resources to enable the proper operation of the service that will assist to overcome the emergency. In this context, Figure 4 illustrates a new situation of the presented scenario in which the municipal authority coordinates from the MANO stack the deployment of a complementary service that is aimed to assist the operations during an emergency (e.g., a vehicle collision). In this case, the main purpose is to enable the communications from the SUAVs to an emergency response team managed by the department whereas the team is moving towards the location of the emergency. Thus, the response team can have a better situational awareness and prepare and coordinate in advance the steps required to mitigate the emergency. To that end, the SUAVs will execute the new functionalities and will be positioned in such a way that they will be able to capture the occurrence and to stream the video content to the response team vehicle (e.g., an ambulance). Moreover, the response team members have access to this situational information due to the functionalities deployed in the vehicle itself in which it travels. For this purpose, the automotive infrastructure provider provision the municipal authority with the NFVI-PoP introduced in Section 3.2.

### 4.3. Network Service Implementation Considerations

This section aims to outline the functionality of the proposed network services in order to demonstrate the potential benefits of the platform in a use case such as the previously commented one. We consider the traffic control situation in which a municipal authority has to extend the service provisioned due to the occurrence of an emergency situation (e.g., a crash between vehicles causing a traffic jam with a high risk of provoking more collisions).

First, it is important to consider that, from the point of view of the municipal authority, one of the design keys to be taken into account when implementing the network service must be the ability to modify the service in real time, and in the most agile and efficient possible manner, in order to address the new requirements that may arise due to a changing event in a scenario such as the one presented here (in which emergency situations can be expected to occur in advance). To this effect, the design of the network service in this experiment is based on a slicing model, where the infrastructure composing the experimental testbed is capable of hosting diverse logical end-to-end networks tailored to fulfil diverse requirements requested by a particular application or service. Furthermore, each slice can be considered as a network service by itself, facilitating the extension of its functionality by the inter-operation with other existing slices. This is possible by means of the use of the NFV technology in each of the infrastructures, allowing the division of the physical resources through virtualization into different logical network components, or VNFs, that will make up each of the slices. This design approach is illustrated in Figure 4, representing each of the VNFs composing each of the slices included by the complete service in a different colour. Next, each of these slices are defined along with their purpose.

On the one hand, depicted in the figure using the violet colour, the service encompasses the slice that is in charge of providing connectivity to the rest of the services that are deployed throughout the municipal authority’s platform. This slice, hereafter referred to as the core-slice, provides its functionality through the multi-site execution of the VNFs called the access router and the 5G core router on the NFVIs supplied by the SUAVs infrastructure and cloud infrastructure providers, respectively. Both VNFs enable a secure transmission of information to the core network hosted by the cloud NFVI over any untrusted non-3GPP access network. For this purpose, the 5G core router VNF implements the user-plane protocol stack defined by 3GPP for a non-3GPP interworking function (N3IWF), and supports the network routing functionalities within the cloud domain. Meanwhile, the access router VNF runs the user-plane protocol stack defined by 3GPP for a 3GPP User Equipment (UE) to reach the core network via an untrusted non-3GPP access. Moreover, this VNF also supports the network routing functionalities to enable the connectivity of the subsequent VNFs and network services accommodated by the SUAVs platform with the core network or Internet.

To endow users with an alternative communications channel in traffic jam situation, reducing the overload and congestion of the existing cellular network, the service considers an additional slice represented in the figure with its composing VNFs coloured in blue. This slice, named as initial-slice, leverages the slice mentioned before (i.e., the core-slice) and addresses a three-fold objective: (1) to supply users situated within the coverage area with an alternative communication channel to browse through the Internet; (2) to enable the transmission of information messages from the municipal authority to those who are connected; and (3) to enable the delivery of real-time video content to the municipal authority with the goal of improving the situational awareness in the context of high traffic density. To accomplish this, the slice deploys, on each SUAV of the SUAVs NFVI, a series of lightweight VNFs that can operate with the VNFs of the other SUAVs through the FANET presented in Section 3.1. These include the Router/AP VNF, which provides a WiFi access point to the end-users, besides the network routing functionalities to support the communications of those users. In this slice there are also the video surveillance VNF, which aims to obtain and disseminate the video content originated by the camera device onboarded on the SUAV to the municipal authority. Finally, our reference use case conceive the instantiation of the so-called flight control VNFs, which are in charge of both the trajectories and the flight plan that each one of the SUAVs hosting its execution must follow.

**Figure 5:**
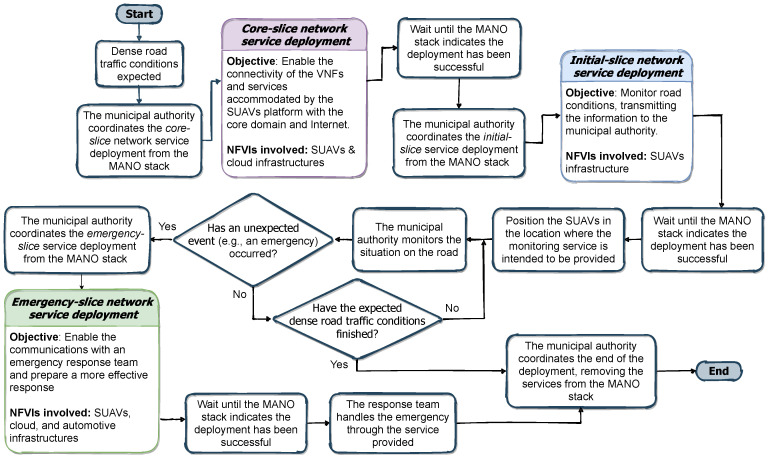
Flowchart of the use case and network service definition.

In the case of an emergency situation, the MANO system included within our framework can coordinate the deployment of an additional slice, which is responsible for providing the appropriate facilities to a response team orchestrated by the municipal authority to deal with the emergency. This slice, referred to as emergency-slice, and highlighted in the figure by colouring the VNFs that make it up in green, covers the deployment of an IP telephony service, isolating the VNFs comprising these services from the VNFs of the previously described slice. As a result, the response team can contact the municipal authority, and even receive video content while approaching the accident scene, with the objective of preparing a more effective response to the emergency. In this case, the slice contains two VNFs not introduced before that are called as IP telephony server and DNS server. These VNFs are responsible for managing the call signalling messages exchanged by the IP phones in order to establish and terminate calls between the response team and the municipal authority, and for providing the name resolution service required by the telephone service, respectively. To this end, this slice carries out the deployment of the VNFs in a multi-site fashion, as shown in the figure, covering the three NFVIs described in the previous section (see Section 3). With the aim of emphasizing the major considerations for implementing each network service encompassed by each of the above mentioned slices, Figure 5 summarizes graphically with a flowchart the most relevant aspects presented during the use case description.

It is important to emphasize that the slicing basis on which the implementation of the global service has been designed, allows a more flexible orchestration of the services provided, being possible to benefit from the discrimination of the resources made available for each one of the functionalities included by means of every slice. For instance, the complete service is designed to be able to simultaneously provide the described functionality of both the initial-slice and the emergency-slice, supporting the traffic exchange through the core-slice. On the opposite, if the latter undergoes an excessive overload and the emergency needs an increase in the number of reserved resources (for example, including an additional video source to have another perspective of the accident), the initial-slice can be removed without affecting the overall functionality of the service managing the emergency.

An additional, relevant aspect to consider within our framework, is the possibility of having periods of intermittent connectivity due to the mobility of the devices utilized, both SUAVs and vehicles. In the latter case, as the vehicle moves along the road, the OBU inside the vehicle will actively check for RSUs in range and which one is the best one available to connect to at that time. Once the OBU identifies the one with the strongest signal, and if it was not already connected, it will perform a handover, which in simple terms means that the OBU connects itself to that RSU. During this process, for a few seconds, there is loss of communication with the infrastructure. In a case where a vehicle goes to a place where there is no RSU coverage, the OBU (as well as all the equipment that is connected to it, i.e., the NFVI) will not have a point of connection and until the vehicle returns to a location with coverage, it will have no connection to the vehicular infrastructure. Likewise, this temporary loss of connectivity means that the VNFs that are deployed on the vehicles will not be able to operate with other VNFs deployed on other vehicles or NFV infrastructures, until connection to the infrastructure is re-established. These were the conclusions reached in [30], where different mobility use cases were presented and explored to evaluate the possible effects of connectivity loss within the vehicular infrastructure, and the impact caused on the operation of the VNFs.

Lastly, in the case of the SUAVs, considering that it is a platform that it is deployed on-demand in the location considered most appropriate to provide a network service, coordinating and controlling the flight plan (i.e, the trajectories to be followed by the mobile devices) at all times by the infrastructure provider, this intermittent connectivity drawback becomes less significant. Thus, it can be assumed that, by controlling that flight plan, SUAVs can be positioned and maintained (e.g., landed on the ground, or in a static hovering situation in the air) in such a way that there is minimal connectivity loss that could affect the operations of the SUAVs infrastructure (including the functionality of the hosted VNFs).

## 5. Implementation and Analysis

This section aims to validate the multi-site orchestration framework using the emergency use case presented in the previous section. First, we present the experimental testbed used in the evaluation process. It should be highlighted that each Infrastructure Provider (5G core and SUAVs, and automotive) are deployed in different countries, namely Spain, in Madrid (hosted by the Universidad Carlos III de Madrid), and Portugal, in Aveiro (hosted by the Instituto de Telecomunicações). By physically separating both sites we are one step closer to the reality, since the ambulance and the SUAVs do not belong to the same network region.

### 5.1. Experimental Testbed

This subsection details the implementation technical aspects of every component comprised within each of the infrastructures included in the testbed, leading to the realization of the proposed framework.

#### 5.1.1. 5G/Cloud Infrastructure Provider

Located in the 5G Telefonica Open Network Innovation Centre (5TONIC) laboratory facilities, the 5G/cloud infrastructure provider plays a leading role within the proposed framework to enable the provisioning of network services in a vertical such as the automotive industry, utilising SUAV devices to support the operations of such services. This infrastructure was created during the realization of one of our prior works [31], which presents the design and construction of a multi-site NFV experimentation platform, aligned with the reference architecture defined by ETSI, and implemented entirely using open-source technologies. For the sake of completeness, in the following lines we include a brief description of its most relevant implementation aspects. Regarding the open-source technologies, the installation of the open source MANO (OSM) release SEVEN [32] tool is particularly noteworthy, since it implements the elements of the ETSI-NFV reference architecture identified as NFV orchestrator (NFVO) and VNF manager (VNFM) to support the orchestration of end-to-end network services in a flexible and automated fashion. This installation has been carried out in a virtual machine within the infrastructure following the recommendation in terms of requirements provided by the OSM documentation (i.e., two central processing units (CPUs), 8 GB of random access memory (RAM), and 40 GB of storage disk).

In addition, this infrastructure integrates two independently-operated cloud computing platforms, offering two separate NFVIs that make it possible to deploy multi-site services, and thus, to host the development of different experimental activities. Likewise, each NFVI is managed and controlled by OpenStack [33], an open-source tool that tailors the functionalities of the element defined as VIM in the ETSI-NFV standard. On the one hand, one of the NFVIs are comprised by elements that supply limited computing capacity in terms of storage, networking and processing, in order to support the development of network services focused on environments beyond the edge, where resources are not as plentiful as they could be in a data-centre environment. For this purpose, this NFVI has 2 mini-ITX computers, each featuring a quad-core processor, 8 GB of RAM and 8 GB ethernet-type network interfaces as computing resources, and leveraging the Kernel-based virtual machine (KVM) solution to support the execution of VNFs in the form of virtual machines. Besides, this first NFVI integrates 10 single board computers model Raspberry Pi 3B+ as compute nodes that have, as technical specifications, a quad-core processing unit, 1 GB of RAM, 1 Gigabit Ethernet port, a 2.4 GHz and 5 GHz IEEE 802.11.b/g/n/ac wireless interface, 4 USB ports that make possible to increase the number of network interfaces by means of adapters, and a 40-pin general purpose input/output (GPIO) header to enable the integration of external hardware elements such as sensors, or LED lights, to extend the available computer functions. In this case, these single board computers allow the deployment of lightweight VNFs (i.e., VNFs that do not demand high computing capacity to be executed) through the container-based virtualization technology offered by Linux (LXC/LXD).

To accommodate more elaborate experimentation, and thus, enable the development of network services composed by the interactivity of more complex functions such as those that may be expected in the core network domain, the 5G/cloud infrastructure provider environment integrates a second NFVI with more powerful and robust computing resources. More specifically, this NFVI comprises three server computers, each one of them providing a computing capacity of 8 CPUs, 32 GB of RAM, and 2 TB of disk storage to deploy VNFs in the form of virtual machines (once again, the solution adopted for this is the KVM-based virtualisation technology). It is worth mentioning that, among the network services that this platform envisages, it includes the development of a 5G core network defined by the 3rd generation partnership project (3GPP) capable of providing connectivity to end-users in a secure manner, whether or not access comes from a 3GPP access network [34,35]. In the latter case, a Non-3GPP inter-working function (N3IWF) would be responsible for providing access to the core network, ensuring confidentiality, integrity and authentication in the course of communications. From this perspective, this NFVI provides the implementation of a basic 5G core network prototype (i.e., there are elements of this 5G core network defined by the 3GPP that are still under development) through the provision of the basic forwarding functionalities defined by the 3GPP for the N3IWF element. Thus, the core network implementation supports the user-plane stack defined by the 3GPP for non-3GPP access networks, making use of the generic routing encapsulation (GRE) and internet protocol security (IPsec) network level protocols with which the 3GPP stipulates this secure access. This enables the connection with the available services that can be offered under the scope of this core network, such as an IP telephony service. In addition, it should be noted that the functionalities described above about 5G core network services have been carried out in the form of VNFs, so that they can be deployed dynamically through the MANO platform integrated in our framework.

Moreover, this infrastructure includes the repository with the implementation of the VNFs that have been presented in Section 4. In this context, Table 1 summarises the most relevant technical implementation aspects of these functionalities, indicating the NFV infrastructures in charge of their execution.

#### 5.1.2. SUAVs Infrastructure Provider

This section is devoted to outlining the key aspects in the implementation of the elements that comprise the NFV SUAV-based infrastructure. As already stated in Section 4.1, this part of the platform is responsible to provide a communications service aimed to assist the operations of a public safety entity (part of a municipal authority) in a context of dense road traffic conditions. Moreover, this platform will undertake the appropriate measures to adapt this service (e.g., change the position of the SUAVs, increase the number of SUAVs to the mission, etc.) in case an emergency occurs.

In the first place, the platform (also located in 5TONIC) is equipped with four SUAV units (model Parrot Bebop 2) that support the deployment of different communications services through the execution of several lightweight VNFs. For this purpose, every aerial unit transport a Single Board Computer (SBC) that supplies the required resources in terms of computation, network, and storage, as well as the virtualization layer. In particular, the Raspberry Pi model 3B+ has been selected as SBC because, in addition to providing the above mentioned computing capabilities, it can easily be on-boarded on top of the SUAVs given its dimensions without compromising the flight operations. As for the technical specifications, these devices feature four CPUs, 1 GB of RAM, 32 GB of storage disk, and two network interfaces: a gigabit ethernet port, and a 2.4/5 GHz wireless card with IEEE 802.11 b/g/n/ac support. This latter is oriented to enable the participation of these devices in the implementation of the FANET described in Section 3.1. Additionally, some of the SBC units integrate a removable wireless adapter (2.4 GHz and IEEE 802.11 b/g/n/ support) in order to enable the creation of wireless access points, and thus, support the access from terrestrial units to the aerial infrastructure. Lastly, the SBCs support the container-based virtualization technology (LXC/LXD) to enable the previously mentioned execution of lightweight VNFs.

To complete this NFV infrastructure, besides the computational aerial units implemented by the SBCs on-boarded over the SUAVs, the GCS includes a mini-ITX computer (with Linux Operating System, 8 CPUs, 8 GB RAM and 125 GB of storage disk) that complements the computational platform, with the objective of hosting the VNFs that require more powerful resources to be executed. For this aim, this node relies on the KVM-based virtualization technology. Moreover, this infrastructure has an additional mini-ITX computer located also in the GCS for the execution of the VIM in charge of governing the resources that comprise the previously mentioned NFV-SUAVs Cloud platform. Similarly to the NFVIs described in the previous section, the open-source software used to manage these hardware resources is OpenStack. Accordingly, the MANO platform can orchestrate the deployment of the functionalities designed for the SUAVs Infrastructure Provider environment in order to implement the services defined for each of the slices included in the use-case scenario.

#### 5.1.3. Automotive Infrastructure Provider

This section focuses on providing the key aspects of how the NFV technology is implemented into the vehicular network. As mentioned in Section 4.1, this part of the platform is responsible to enable the communication between the distinct sites via the RSUs, as well as to provide the ability to host the required virtual functions on the vehicles themselves, thus bringing more flexibility to the overall solution.

To incorporate the NFV technology into the vehicular network used in this work (see Section 3.2) in such a way that no major changes are required to the way it is designed and to the way it operates, the use of virtual overlay networks is required. These virtual networks sit on top of the already existing vehicular network and are responsible for connecting all of the solution’s NFV components and thus allowing communication between them. To implement the virtual networks, Virtual Extensible LAN (VXLAN) technology was used, as it enables the creation of a single logical network for machines located at different networks, as it is the case with the vehicular network. As for the on-boarded hardware, the choice came down to the Raspberry Pi, as it is a simple lightweight SBC which supports the virtualization required by the NFV technology while at the same time being able to be on-boarded on the vehicles without any problems. In order to integrate them into the VANET, each one is connected to an OBU, and a network is created between them, where the OBU acts as a the network’s gateway, routing the on-boarded hardware’s traffic into the scope of the vehicular network, thus allowing it to reach the Internet (and thus the VIM) using the VANET’s infrastructure.

In terms of the equipment used in this platform, the VANET used in this work is comprised of an LMA and a number of RSUs and OBUs. The RSUs and OBUs are implemented in NetRiders from Veniam (www.veniam.com), which are SBCs running a custom Linux based Operating System (OS) and are equipped with IEEE 802.11p, 802.11b/g/n and cellular interfaces. The LMA runs on a dedicated machine (running a Linux based OS). To support the deployment of the lightweight VNFs, each vehicle will carry extra hardware alongside the OBU, namely Raspberry Pis model 3B. These SBCs have the required resources and the ability to host the virtual functions by supporting container virtualization via the use of Linux Containers (LXC). Some of them have additional wireless interfaces to allow the creation of wireless access-points (APs), and thus enable end-users to interact with the virtual services running on the whole platform. Moreover, to complement the infrastructure and to enable the deployment of virtual functions that are more demanding in terms of resources, there is also a laptop (running Ubuntu) which supports KVM virtualization. Finally, the remaining components of the NFV infrastructure (such as the VIM) are hosted on a dedicated machine running a Linux based OS.

### 5.2. Practical Evaluation: Deployment Times Profiling

With the aim of verifying the ability of the experimental platform to adapt to emergency situations, such as the one presented before, we have analysed the time required in our environment to carry out the deployment of the services that will be executed throughout this use case, especially focusing on the emergency service.

In this context, we have carried out the measurements of the deployment time that the first slice (i.e., the so-called core-slice) takes to deploy the service it comprehends. Accordingly, the deployment has been coordinated from the OSM stack, measuring the time it takes to consider the success in completing the deployment. To calculate the deployment time, OSM provides a timestamp corresponding to the instant at which the deployment of the network service is initiated, and another timestamp of the instant when the deployment is completed. In this context, OSM considers that a deployment is completed when all the VNFs composing the network service have been instantiated in their corresponding NFVI, and correctly configured to provide the expected functionality. With this information, by subtracting both timestamps, the time taken for the service to be deployed can be obtained. Once the deployment time is calculated, the network service is removed from the testbed so as to ensure the initial conditions in subsequent deployments. In order to conduct a significant study from a probabilistic perspective, we have repeated this process 30 times. It is worth noting that the same conditions have been preserved for each of the 30 iterations carried out in the deployment of the service corresponding to the slice. These conditions encompass the realization of each deployment in a controlled laboratory environment, with the SUAVs placed at 5TONIC laboratory (located in Madrid, Spain), and with the vehicle emulating the ambulance by means of an OBU at the laboratory of the Instituto de Telecomunicações (located in Aveiro, Portugal). In addition, both the SUAVs and the vehicle have been kept in a stationary state (i.e., the SUAVs landed in the ground, maintaining the same position, and the vehicle with no movement).

**Figure 6:**
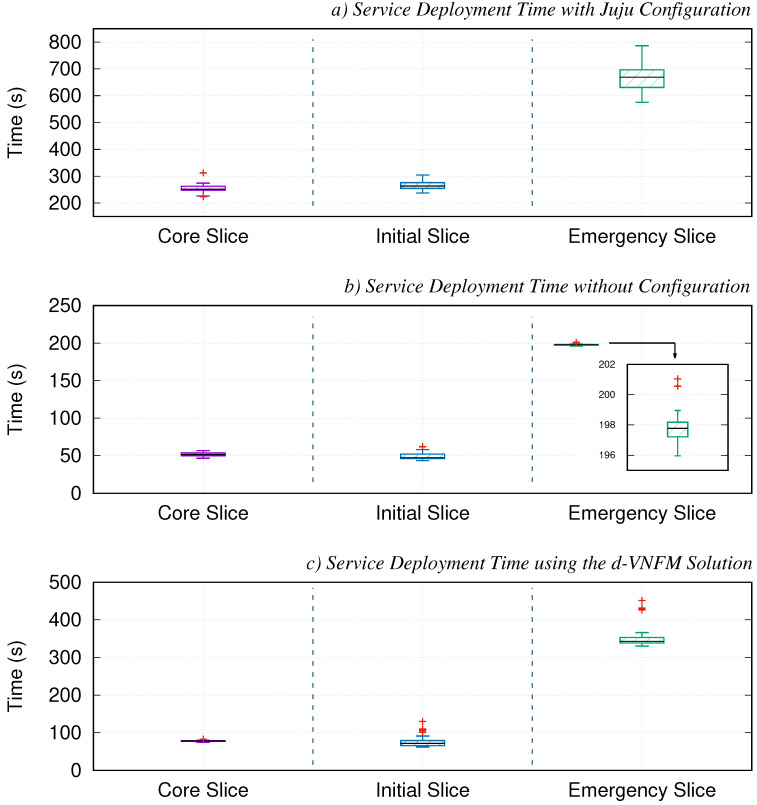
Service deployment time measurements of each slice.

As illustrated in Figure 6a, the values of this experiment have been represented using a box-and-whisker plot, which allows to visualize at a glance the time-series data obtained in relation to the deployment times. Particularly for this slice, it can be seen that the deployment time is gathered around 250 s (median value), with 226 and 274 being the values of the lower and upper quartiles (usually identified as Q1 and Q3), respectively. The median value, or also known as Q2 in this type of graph, is represented by a horizontal black line. Using Q1 and Q3, the interquartile range (IQR) can be obtained, which multiplied by the well-known factor 1.5, allows to calculate the maximum length that the whiskers of the representation will have from the values Q1 downwards, and Q3 upwards. Likewise, this allows the identification of outliers (represented by a red cross), which are those values that are beyond the end of the whiskers (both upper and lower).

To determine which is the time required to deploy the second slice, referred to as initial-slice in this work, we have performed from the OSM stack a similar procedure to the one described above, with the peculiarity that in this case, an instance of the service comprised by the core-slice was present in each one of the iterations deploying this new service. Thus, it is possible to study whether the fact of having already a service allocated within the testbed can affect the performance of future deployments. In this case, Figure 6a shows that the deployment time for the 30 runs is between 237 and 304 s. This is a very similar result to the one obtained during the previous experimentation block, since both slices have the same number of VNFs composing the service. Considering this, it can also be inferred that the presence of a previous deployment allocated within the testbed does not really affect the deployment time of the following services.

Continuing the experimentation process, and using the defined methodology, we proceeded to calculate the deployment time of the service encompassed by the slice identified as emergency-slice. In this experimentation block, each of the iterations includes an instance of the services covered by the core-slice and the initial-slice before proceeding to the deployment time profiling of the emergency-slice. Figure 6a in this case shows a noticeable increase in relation to the deployment time compared to the previous slices. In particular, the time to deploy the service in charge of addressing the emergency situation has a median value of 668 s, with a maximum spread in between 575 and 780 s. In other words, it can be seen that the service can even take around 13 minutes to be deployed. Considering that both SUAVs and vehicle NFVIs have similar accesible resources, and the provision of services using a set of standardized operations through the utilization of NFV technologies in the framework, this increase is due to the fact that the number of VNFs included in the emergency slice is larger (specifically, the service has six VNFs against the two included in each of the other slices).

Although the time may seem reasonable in terms of comparison with the reference values set for service deployment for the 5G era [36], the upward trend observed during the experiments due to the number of VNFs comprised within the service, leads to consider if the proposed framework has the sufficient capability to agilely and flexibly be adapted in scenarios where the service requires greater complexity, with a larger number of VNFs, to manage an unexpected situation. Indeed, there are emergency response teams such as SAMUR (the municipal emergency service in Madrid) which have an average emergency response time of about nine and a half minutes [37], what indicates that if a service such as the one proposed here were intended to be deployed, the team would have to initiate the emergency coordination without the support of the designed communications service.

With the objective of identifying at what stage of the deployment this time can be reduced, we have carefully analysed how the MANO platform operates throughout the instantiation of a network service. Before this analysis, it is important to remark that the MANO platform within our framework is based on the Open Source MANO Release SEVEN (see Section 5.1), which implements the different components of a MANO platform using a cloud-native model based on Docker containers, and provides a Kafka bus to enable the interaction of the different elements/containers with each other in order to automate the deployment of end-to-end network services. In this respect, once the instantiation process is executed, OSM consecutively undertakes the following phases:

1. Processing of information uploaded to the MANO platform with both the network service descriptor (NSD) and the descriptors of the virtualised functions (VNFDs) that comprise it. In this first phase, the MANO platform determines which ones of the VNFs require configuration by the VNFM element included in OSM, implemented through Juju.
2. Coordination with the different VIM entities configured in the MANO platform responsible for managing and allocating the resources that will be used by the VNFs in each of the corresponding infrastructures where they are meant to be executed. Simultaneously, the MANO platform at this stage reports Juju to start the preparation of the VNF configuration environment, and for this purpose, Juju runs a series of Linux containers within the host in charge of executing OSM. In this context, a container is created for every VNF that require configuration, associates itself with one of those VNFs, and Juju carries out the appropriate software installation to enable the subsequent configuration of the associated VNF. Moreover, this is possible through the execution of a set of Juju scripts called proxy charms, which enable the use of the Ansible playbooks technology by means of the base charm layer contributed within the OSM software [38].
3. Once OSM is notified by the VIMs about the proper instantiation of each image of every VNF (i.e., the softwarization unit loaded into the VIM with pending configuration to provide specific functionality) has been correctly carried out, it also collects the information regarding the management IP address that allows the configuration of these VNFs. Next, OSM transmits this information to Juju to proceed with the configuration tasks, executing the specified actions for each VNF in their corresponding container.
4. Finally, when each container completes the configuration activity of its associated VNF, Juju sends a message to OSM to inform that the configuration process has been completed. Once OSM processes this message, the deployment of the service is considered complete.

To ensure that this process is as efficient as possible, OSM processes in parallel both the instantiation and configuration stages defined for phases 2 and 3. Despite this, as we observed in one of our previous works [31], this process requires such a high processing load that it results in a significant delay to the service deployment time. Furthermore, in this work we could appreciate how this delay is also accentuated by the complexity of the synchronisation tasks between Juju, the Linux containers deployed by the own Juju, and OSM to carry out the configuration and the lifecycle management of every instantiated VNF. In the light of the lessons learned from our previous work, and in order to confirm that the configuration stage is a possible candidate for improvement to reduce the service deployment times, we proceeded to measure the time taken to deploy the services contained in each one of the slices, but this time without configuration. To do this, the same methodology mentioned above has been repeated, consecutively instantiating each service without configuration 30 times. In this case, Figure 6b depicts how these times are indeed far below from what was seen in the previous set of tests, and how the VNF configuration increases the deployment time by, at the very least, a factor of 3. The following section elaborates on the development carried out in the scope of this work to mitigate this factor within the proposed framework.

### 5.3. Publish–Subscribe Configuration Function

In order to overtake the previously analysed limitation in terms of service deployment time, this section presents our developed and integrated solution within the overall experimental platform. This solution intends to decouple the configuration stage from the OSM stack, supplied by Juju, and to carry out an alternative implementation of the functionality specified for the element VNFM of the ETSI NFV architectural framework.

One of the design keys of this solution is that it considers the decentralization of the VNF configuration function so that it does not result in a processing overload for the host executing the MANO stack (in our case, the machine hosting the OSM software). In particular, the solution proposes to localize an instance of the novel VNFM implementation in each one of the NFVIs integrated in the MANO stack, and to place it close to the VIM in charge of the resources of this infrastructure. In this context, each instance will be only and exclusively responsible for the configuration of those VNFs allocated by the infrastructure where it is located.

An additional key aspect to consider about our solution is that it is based on a publish–subscribe model. The main attribute of this model, which is widely spread in the Internet of Things environments [39,40,41,42], is that it allows the exchange of information related to events in an efficient and asynchronous manner. To that end, the model defines topics based on the use of identifiers, that will be in charge of categorizing and organizing the information flow related to different events. With these identifiers, the elements called within the model as publishers can produce or publish information referring to a specific event through its predefined topic. Subsequently, this information will be consumed by those elements subscribed to the topic under consideration. To enable both, the model also defines the figure of the broker, which is in charge of receiving the information published under a topic, storing it in a persistent manner, and asynchronously distributing it to subscribers.

**Figure 7:**
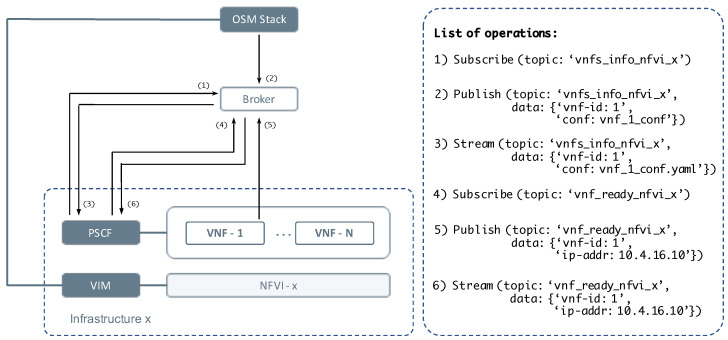
Flow diagram of VNF configuration solution based on the publish–subscribe model.

Based on this perspective of a publish–subscribe model, our solution, hereafter referred to as the publish–subscribe configuration function (PSCF), proposes the use of this model to synchronise the different elements that come into play during the configuration stage of a network service and to coordinate the configuration activities. To do so, an initial information flow has been defined to be published at the time of deployment by the MANO stack, containing the configuration data of each of the VNFs comprised by the network service. This first information flow is also structured in groups in which the information contained corresponds to the VNFs that are going to be deployed on the same infrastructure, using for this purpose a topic per infrastructure when publishing (for instance, ‘vnfs_information_nfvi_x’). Thus, each PSCF could subscribe to the appropriate topic and only receive the precise information for the configuration of the VNFs under its charge (i.e., those allocated within its particular NFVI). In addition, the data published under a topic like the one previously mentioned, includes the VNF identifier (for example id = ‘vnf_1’) and the set of configuration actions for the associated VNF, so that the PSCF is able to process and collect the configuration information to proceed with a subsequent VNF configuration. On the other hand, once the PSCF has the necessary information to carry out the configuration of the VNFs, it has to realize when these VNFs are ready to be configured. For this purpose, a new information flow has been defined to be shared following the publish–subscribe model, in which each VNF publishes when it is active (i.e., when it has been completely instantiated). Again, a topic has been defined for each infrastructure (e.g., ‘vnf_ready_nfvi_x’), including as data under the publication the VNF identifier, along with the management IP address that enables the communications between the VNF and the PSCF in order to receive the configuration commands (e.g., ‘vnf-id: 1; ip-address:10.4.16.10’). This information can be consumed by the PSCF after prior subscription to the topic, allowing to start the configuration actions defined by the first information flow, and thus carry out the configuration assignment. Figure 7 summarizes this synchronization process to perform the VNF configuration proposed by our solution.

To carry out the implementation of a preliminary version of the PSCF and validate the viability of the proposed solution, a virtual machine has been configured within the segment of the experimental testbed denominated as Cloud infrastructure provider, installing the necessary software to develop the required functionalities within the publish–subscribe model, as well as those required to carry out the configuration of the VNFs. On the one hand, the machine is supplied with the Kafka open-source software [43], which allows the streaming of events based on the publish–subscribe model, implementing the role defined within the model for the figure of the broker. This software enables a communications bus, denoted as Kafka bus, that receives the information flow of the events produced by the publishers (organised as mentioned above by means of topics), and makes it available to those consumers that are subscribed. Additionally, this machine incorporates the python library kafka-python, implementing a client of the application programming interface (API) provided by the Kafka to interact with its bus. This library is used in the PSCF for the creation of a script that allows to consume the messages published on the bus, processing them to gather the pertinent information, and executing the configuration commands of each VNF in parallel (once the message corresponding to the completion instantiation VNF has been received).

On the other hand, to carry out the configuration function of the PSCF once the corresponding message has been consumed and processed, the machine has been provided with the installation of an Ansible server [44], which is a technology that allows the automated provisioning of software and its configuration management for deploying applications. Furthermore, Ansible provides a functionality that makes possible to describe and group the sequence of configuration actions for one or more machines to be carried out by the Ansible server through what is known as Ansible playbooks. These playbooks are specified in .yaml files so that they can be reused as often as necessary. In our solution, each VNF has an associated playbook that is loaded in the OSM platform through the VNFDs. These VNF playbooks conform the first information flow that has been defined to be streamed through the publish–subscribe model of our solution. To simplify this process, and thus avoid altering the OSM software stack, the playbooks of each VNF have been manually pre-loaded in the PSCF machine. In this manner, the PSCF will proceed to execute these playbooks (as mentioned above, in parallel), once it consumes the message published by each VNF specifying that it is ready. In this sense, each VNF includes a python script that is executed in the machine startup, and with which, through the mentioned library kafka-python, publishes the message reporting that it has been instantiated in the Kafka bus.

With this initial version of our PSCF, we have carried out the deployment of the services contained by each slice, and measured the time it takes to complete the whole process (i.e., instantiation plus configuration). As it can be seen in Figure 6c, this time is quite lower than the one obtained in the previous scenario with the configuration made by OSM through Juju. This confirms the viability of our solution. In fact, these results have been collected by simply having one instance of PSCF. Hence, it can be perfectly assumed that these values can even be better by distributing one instance of our PSCF per each NFVI, as indicated in the design keys of our solution.

### 5.4. Video Service

In this section we corroborate that the overall service can offer the expected functionality by means of the interoperation between the three slices. In this context, this deployment has been done using the development of the PSCF for configuring each VNF. Thus, in the case of an emergency (e.g., a collision between vehicles), the municipal authority can become aware of the emergency and effectively coordinate the required operations for handling the emergency together with the public safety department. For this purpose, our use case contemplates the option of enabling the simultaneous streaming of different real-time video feeds. In particular, the first video feed enables to monitor a section of road (known in advance as a risk or conflict zone), improving the situational awareness on the part of the corresponding municipal authority. From this video content, the municipal authority detects that an emergency has occurred and the scenario includes an additional real-time video feed to capture in detail the occurrence of the emergency. Then, this additional video content is streamed to the response team that is on its way, to allow the definition of an action plan, prioritising the most urgent actions.

In this context, once the services of the slices have been deployed, and correctly configured, an evaluation of the network performance supported by the interoperation of these services has been carried out with the aim of proving the stable and flawless operation of the video service explained. On the one hand, the network performance available for the video content of the initial-slice service has been evaluated. This video content will be retransmitted from the VNF offering a wireless access point allocated by the SUAVs NFVI, to the municipal authority (see Figure 4). In order to analyse whether the network performance can support that video content, the available bandwidth between the mentioned endpoints has been measured using the Iperf tool [45], and the round trip time (RTT) with the Ping tool [46]. The evaluation results for this scenario indicate an available bandwidth of 21.20 Mbps, and an RTT of about 7 ms. From the latter value, it can be inferred that the end-to-end delay performance is about 3.5 ms. These outcomes comfortably comply with the requirements defined by some relevant organizations within the telecommunications sector for this type of real-time video services. In particular, the ITU-T recommends end-to-end delays below 150 ms so that the display at the destination is not significantly affected [47]. Moreover, the 3GPP technical specification [48] in charge of defining the key performance indicators (KPIs) for the SUAVs operations within the 5G communications systems, indicates that the available bandwidth during the streaming of real-time video must be at least 0.06 Mbps, and that the end-to-end delay cannot exceed 100 ms. As can be noted, this specification is even more restrictive in terms of end-to-end delays than the ITU-T recommendation. In addition to this, the obtained results also manifest that the transmission of a high-definition (HD) quality video content (which requires a bandwidth of at least 5 Mbps for a resolution of 720p) is feasible.

With respect to the emergency scenario, the same analysis has been carried out but this time between the endpoints that allow the video content streaming with the purpose of managing the emergency situation, i.e., between the VNF providing an access point allocated for the emergency-slice by the SUAVs NFVI, and the wireless access point that will be used by the response team, allocated within the ambulance vehicle of the automotive NFVI (both functionalities are part of the service implemented by the emergency-slice). As in the previous scenario, notwithstanding that the results are limited by the wireless communications technology of the automotive platform (i.e., WAVE/IEEE 802.11p), the requirements mentioned above for supporting real-time video streaming are sufficiently fulfilled since the results provide an available bandwidth of 6.85 Mbps, and an RTT of almost 40 ms (or in other words, the maximum end-to-end delay never exceeds the 20 ms). In the case of the latter, the value is increased with respect to the previous scenario, since the points used for the aforementioned measurement are located in different geographical points. In fact, they are in different countries: the former is in Madrid, Spain, and the latter in Aveiro, Portugal.

**Figure 8:**
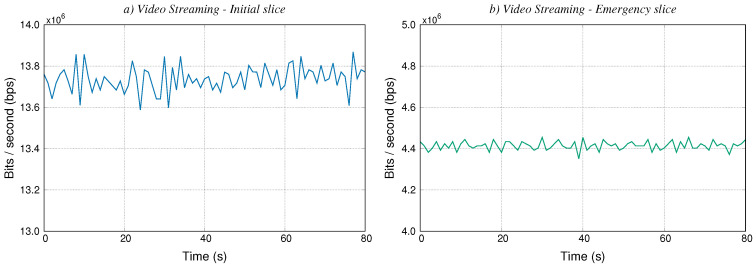
Traffic flows of the video service.

Given that the network performance evaluation has demonstrated the feasibility of supporting the video service, the outlined real-time videos are streamed. For this purpose, the Iperf tool has been used again, since it allows to transmit an UDP traffic stream at a specific data-rate to emulate the video content stream. As can be seen in Figure 8, with respect to the first video content, a rate of around 13 Mbps has been selected to emulate a very high-definition quality video that allows a wide stretch of road to be monitored in the finest possible detail. According to the second video content, a high-definition quality video has been emulated, lower than the previous one, so that it can be transmitted to the response team’s ambulance. Furthermore, it can be seen that the service offered supports the simultaneous transmission of both contents.

## 6. Conclusions

Based on the lessons learned from two lines of research aimed at adapting the NFV technology to environments with mobile devices, namely SUAVs and vehicles, this paper analyses the synergies that can be exploited if these two research lines intersect. In this context, this work presents a novel framework that considers the joint integration of three types of NFV infrastructures, with the aim of creating a distributed and more complete NFV ecosystem capable of supporting the flexible deployment of vertical services.

Particularly, these infrastructures are represented by different computing resources and capabilities, which means that the framework can orchestrate very diverse and useful services, taking advantage of the characteristics of each of the infrastructures that constitute the framework. This can be achieved as the above-mentioned integration allows to extend the functionality of the services provided through the interoperation of the VNFs hosted across the different infrastructures. Furthermore, a complex use case involving the public safety vertical is also defined throughout the work in which the practicality and the potential benefits of the proposed framework are underlined. From this point of view, this use case defines how, in a complex road situation managed by a municipal authority, the infrastructure included in the framework can deploy, in a flexible and automated way, a communications service to provide a road condition surveillance. Moreover, this scenario shows how the service can be adapted in the event of an emergency, so that the functionalities offered by this infrastructure are geared towards managing this problematic situation. Nonetheless, many other services regarding the intelligent transportation systems topic could be addressed and leveraged by the means of the framework here presented, encompassing FANETs as demonstrated here, or any other mobile networks.

On the other hand, the applicability of the proposed framework, orchestrating different network services aggregated in different slices, with the objective of tailoring a video streaming service that allows the municipal authority to coordinate the response operations, has been discussed. We have detected that the process followed in the deployment of the services may not be properly adapted in terms of deployment times to an emergency situation. This is caused due to the task of configuring VNFs to supply their desired functionality. To address this drawback, the work includes an innovative solution of the NFV architectural component in charge of the VNF configuration (i.e., the VNFM) based on a publish–subscribe model and its distribution along each infrastructure.

Future work aims to improve the infrastructure with a more extended set of VNFs for different scenarios. In the short-term, this work also aims at evaluating lighter solutions that are designed to be able to apply virtualization in constrained environments with restricted computing resources, as in the case of the vehicles and SUAVs NFV infrastructures. In addition, further future line intends to dynamically integrate different devices such as smartphones, tablets or laptops into an NFV infrastructure that can be deployed on demand in a particular location to provide a network service. In this manner, NFV infrastructures such as the one presented in this work composed by SUAVs, could flexibly extend their computing capacity with different devices that can be located in the deployment area where a service is intended to be supplied. Finally, the latency of the deployment is another avenue of work, to address the low-latency scenarios envisioned in 5G and beyond.

## Figures and Tables

**Table 1:** VNFs technical implementation details.

VNF (NFVI-PoP)	Brief Description of Functionality	Technical Requirements	Featured Software
5G Core Router (5G/Cloud Infrastructure Provider)	Implementation of the user-plane protocol stack of a 3GPP N3IWF, as well as routing functionalities towards external networks	Prototyped as a VM, using Ubuntu 16.04; 2 vCPUs, 1 GB RAM, 5 GB storage	Linux ip-gre ip-forwarding modules, and the ipsec-tools package
IP Telephony Server (5G/Cloud Infrastructure Provider)	Provide the functions of an IP Telephony service based on the SIP protocol (i.e., proxying of call signalling messages and user registration)	Prototyped as a VM, using Ubuntu 16.04; 1 vCPU, 1 GB RAM, 5 GB storage	Kamailio, an open-source SIP server (Linux package)
DNS (5G/Cloud Infrastructure Provider)	Support a name resolution service, to enable user identification in a functional IP telephony service	Prototyped as a VM, using Ubuntu 16.04; 1 vCPU, 1 GB RAM, 5 GB storage	Dnsmasq, an open-source DNS server (Linux package)
Access Router (SUAVs/Automotive Infrastructure Provider)	Implementation of the user-plane protocol stack of a 3GPP UE, providing access to 5G core network via an untrusted non-3GPP access	Prototyped as a VM, using Ubuntu 16.04; 1 vCPU, 1 GB RAM, 5 GB storage	Linux ip-gre and ip-forwarding modules, and the ipsec-tools package
Router/AP (SUAVs/Automotive Infrastructure Provider)	Implementation of a Wi-Fi access point, supporting the assignment of IP addresses using DHCP, and routing functions	Prototyped as LXC container, using Ubuntu 16.04; 1 vCPU, 128 MB RAM, 4 GB storage	Linux ip-forwarding module and isc-dhcp-server package

## References

[1] 5G Infrastructure Public Private Partnership (2017). View on 5G Architecture (Version 2.0). 5G PPP Architecture Working Group, Whitepaper. https://5g-ppp.eu/wp-content/uploads/2018/01/5G-PPP-5G-Architecture-White-Paper-Jan-2018-v2.0.pdf.

[2] 5G Infrastructure Association (2015). The 5G Infrastructure Public Private Partnership: The Next Generation of Communication Networks and Services. 5G PPP, 5G Infrastructure Association, Whitepaper. https://5g-ppp.eu/wp-content/uploads/2015/02/5G-Vision-Brochure-v1.pdf.

[3] Elayoubi S.E., Bedo J., Filippou M., Gavras A., Giustiniano D., Iovanna P., Manzalini A., Queseth O., Rokkas T., Surridge M. (2017). 5G Innovations for New Business Opportunities. 5G PPP, 5G Infrastructure Association, Whitepaper. https://hal.inria.fr/hal-01488208/document.

[4] Ateya A.A., Muthanna A., Makolkina M., Koucheryavy A. Study of 5G services standardization: Specifications and requirements. Proceedings of the 2018 10th International Congress on Ultra Modern Telecommunications and Control Systems and Workshops (ICUMT).

[5] Banchs A., Gutierrez-Estevez D.M., Fuentes M., Boldi M., Provvedi S. (2019). A 5G mobile network architecture to support vertical industries. IEEE Commun. Mag..

[6] De la Oliva A., Li X., Costa-Perez X., Bernardos C.J., Bertin P., Iovanna P., Deiss T., Mangues J., Mourad A., Casetti C. (2018). 5G-TRANSFORMER: Slicing and orchestrating transport networks for industry verticals. IEEE Commun. Mag..

[7] Nogales B., Sanchez-Aguero V., Vidal I., Valera F. (2018). Adaptable and Automated Small UAV Deployments via Virtualization. Sensors.

[8] Nogales B., Vidal I., Sanchez-Aguero V., Valera F., Gonzalez L.F., Azcorra A. (2019). Automated Deployment of an Internet Protocol Telephony Service on Unmanned Aerial Vehicles Using Network Functions Virtualization. Jove (J. Vis. Exp.).

[9] Gonzalez L.F., Vidal I., Valera F., Nogales B., Sanchez-Aguero V., Lopez D.R. (2019). Transport-Layer Limitations for NFV Orchestration in Resource-Constrained Aerial Networks. Sensors.

[10] Sanchez-Aguero V., Valera F., Nogales B., Gonzalez L.F., Vidal I. (2019). VENUE: Virtualized Environment for multi-UAV network emulation. IEEE Access.

[11] Silva M., Luís M., Sargento S. Edge Virtualization in Multihomed Vehicular Networks. Proceedings of the 10th Workshop on Management of Cloud and Smart City System, IEEE International Symposium on Computer and Communications (ISCC).

[12] Luís M., Gomes C., Sargento S., Ortiz J., Santa J., Fernández P.J., Gil Pérez M., Martínez Pérez G., Barmpounakis S., Alonistioti N., Mouftah H., Erol-Kantarci M., Sorour S. (2020). Exploring Cloud Virtualization over Vehicular Networks with Mobility Support. Connected and Autonomous Vehicles in Smart Cities.

[13] Silva A.P., Tranoris C., Denazis S., Sargento S., Pereira J., Luís M., Moreira R., Silva F., Vidal I., Nogales B. (2019). 5GinFIRE: An end-to-end open5G vertical network function ecosystem. Ad Hoc Networks.

[14] 5TONIC The leading European laboratory in 5G. https://www.5tonic.org.

[15] IT-Instituto de Telecomunicações. https://www.it.pt.

[16] Al-Sultan S., Al-Doori M., Al-Bayatti A. (2014). A comprehensive survey on vehicular Ad Hoc network. J. Netw. Comput. Appl..

[17] Kreutz D., Ramos F.M., Verissimo P.E., Rothenberg C.E., Azodolmolky S., Uhlig S. (2014). Software-defined networking: A comprehensive survey. Proc. IEEE.

[18] Jain R., Paul S. (2013). Network virtualization and software defined networking for cloud computing: A survey. IEEE Commun. Mag..

[19] Yousaf F.Z., Bredel M., Schaller S., Schneider F. (2017). NFV and SDN—Key Technology Enablers for 5G Networks. IEEE J. Sel. Areas Commun..

[20] Sun J., Liu F., Ahmed M., Li Y., Zeng H. (2019). A Unified Framework for Software Defined Sensing, Transmission and Computing. IEEE Access.

[21] Moura J., Hutchison D. (2020). Modeling cooperative behavior for resilience in cyber-physical systems using SDN and NFV. Appl. Sci..

[22] Hussain R., Son J., Eun H., Kim S., Oh H. Rethinking Vehicular Communications: Merging VANET with cloud computing. Proceedings of the 4th IEEE International Conference on Cloud Computing Technology and Science Proceedings.

[23] Zingirian N., Valenti C. Sensor clouds for Intelligent Truck Monitoring. Proceedings of the 2012 IEEE Intelligent Vehicles Symposium.

[24] Zhu M., Cao J., Cai Z., He Z., Xu M. (2016). Providing flexible services for heterogeneous vehicles: An NFV-based approach. IEEE Netw..

[25] Zeng Y., Wu Q., Zhang R. (2019). Accessing from the sky: A tutorial on UAV communications for 5G and beyond. Proc. IEEE.

[26] Khan M.A., Qureshi I.M., Khanzada F. (2019). A hybrid communication scheme for efficient and low-cost deployment of future flying ad-hoc network (FANET). Drones.

[27] Faraci G., Grasso C., Schembra G. (2020). Design of a 5G network slice extension with MEC UAVs managed with reinforcement learning. IEEE J. Sel. Areas Commun..

[28] (2014). ETSI GS NFV 002 V1.2.1. Network Functions Virtualization (NFV); Architectural Framework, European Telecommunications Standards Institute. https://www.etsi.org/deliver/etsi_gs/NFV/001_099/002/01.02.01_60/gs_NFV002v010201p.pdf.

[29] 5G Infrastructure Public Private Partnership (2014). 5G Automotive Vision. 5G PPP, Whitepaper. https://www.crunchbase.com/organization/5g-infrastructure-public-private-partnership.

[30] Silva M., Luís M., Sargento S. Edge Virtualization in Multihomed Vehicular Networks. Proceedings of the 2020 IEEE Symposium on Computers and Communications (ISCC).

[31] Vidal I., Nogales B., Valera F., Gonzalez L.F., Sanchez-Aguero V., Jacob E., Cervelló-Pastor C. (2020). A Multi-Site NFV Testbed for Experimentation With SUAV-Based 5G Vertical Services. IEEE Access.

[32] ETSI Open Source MANO (OSM). https://osm.etsi.org.

[33] Ameixa R., Barrett C., Bonell M., Britten T., Cacciatore K., Huang J., Kloeker F., Kumar A., Lamourine M., Prüßmann G. (2017). Designing, Migrating and Deploying Applications: A Guide to Cloud Applications on OpenStack.

[34] (2019). Standard 3GPP TS 23.501, version 16.2.0. System Architecture for the 5G System; Stage 2. 3rd Generation Partnership Project Technical Specification. https://www.etsi.org/deliver/etsi_ts/123500_123599/123501/15.03.00_60/ts_123501v150300p.pdf.

[35] (2019). Standard 3GPP TS 23.502, Version 16.2.0. Procedures for the 5G System; Stage 2. 3rd Generation Partnership Project Technical Specification. https://www.etsi.org/deliver/etsi_ts/123500_123599/123502/15.02.00_60/ts_123502v150200p.pdf.

[36] 5G Infrastructure Public Private Partnership (2013). Creating a Smart Ubiquitous Network for the Future Internet. 5G PPP, Advanced 5G Network Infrastructure for the Future Internet, Whitepaper. https://5g-ppp.eu/wp-content/uploads/2014/02/Advanced-5G-Network-Infrastructure-PPP-in-H2020_Final_November-2013.pdf.

[37] Ayuntamiento de Madrid (2020). SAMUR—Protección Civil, Atención Sanitaria de Urgencias. https://t.ly/2ZRT.

[38] ETSI Open Source MANO: Example of VNF Charms, OSM Wiki. https://osm.etsi.org/wikipub/index.php/Example_VNF_Charms.

[39] Pham V.N., Nguyen V., Nguyen T.D., Huh E.N. (2020). Efficient Edge-Cloud Publish/Subscribe Broker Overlay Networks to Support Latency-Sensitive Wide-Scale IoT Applications. Symmetry.

[40] Lv P., Wang L., Zhu H., Deng W., Gu L. (2019). An IoT-oriented privacy-preserving publish/subscribe model over blockchains. IEEE Access.

[41] Duan L., Sun C.A., Zhang Y., Ni W., Chen J. (2019). A comprehensive security framework for publish/subscribe-based IoT services communication. IEEE Access.

[42] Wirawan I.M., Wahyono I.D., Idfi G., Kusumo G.R. Iot communication system using publish-subscribe. Proceedings of the 2018 International Seminar on Application for Technology of Information and Communication.

[43] Kreps J., Narkhede N., Rao J. Kafka: A distributed messaging system for log processing. Proceedings of the NetDB.

[44] Red Hat Ansible (2017). Ansible in Depth. Red Hat, Whitepaper. https://www.ansible.com/hubfs/pdfs/Ansible-InDepth-WhitePaper.pdf.

[45] Tirumala A., Cottrell L., Dunigan T. Measuring end-to-end bandwidth with Iperf using Web100. Proceedings of the Passive and Active Measurement Workshop.

[46] Matthews W., Cottrell L. (2000). The PingER project: Active Internet performance monitoring for the HENP community. IEEE Commun. Mag..

[47] (2003). ITU-T Recommendation G.114. Series G: Transmission Systems and Media, Digital Systems and Networks, International Telecommunication Union. https://www.itu.int/rec/T-REC-G/en.

[48] (2020). Standard 3GPP TS 22.125 Version 17.2.0. Technical Specification Group Services and System Aspects: Unmanned Aerial System (UAS) support in 3GPP. 3rd Generation Partnership Project Technical Specification. https://portal.3gpp.org/desktopmodules/Specifications/SpecificationDetails.aspx?specificationId=3545.

